# Long noncoding RNA DSCR8 promotes the proliferation of liver cancer cells and inhibits apoptosis via the miR-22-3p/ARPC5 Axis

**DOI:** 10.7150/jca.79475

**Published:** 2023-01-01

**Authors:** Jiu-Ning Huang, Hui-Ming Zhang, Jun-Dong Cai, Wu-Long Wang, Ping Wang

**Affiliations:** 1 Department of Radiation Oncology, Tianjin Medical University Cancer Institute and Hospital, National Clinical Research Center for Cancer, Tianjin Key Laboratory of Cancer Prevention and Therapy, Tianjin's Clinical Research Center for Cancer, Tianjin, China.; 2 Department of Radiation Oncology, Yantai Affiliated Hospital of Binzhou Medical University, Yantai, China.; 3 Department of Surgery, Tianjin Hospital, Tianjin, China.

**Keywords:** Competing endogenous RNA, Liver cancer, lncRNAs, MicroRNA, Prognosis, Proliferation, Apoptosis

## Abstract

Emerging evidence shows that long noncoding RNAs (lncRNAs) play a vital role in the tumorigenesis and development of cancer, implying that some lncRNAs could be potential therapeutic targets. In this study, we employed Gene Expression Omnibus (GEO) and The Cancer Genome Atlas (TCGA) databases to construct a ceRNA network by bioinformatic analysis, and the Down syndrome critical region 8 (lncRNA_DSCR8)/miR-22-3p/actin-related protein 2/3 complex subunit 5 (ARPC5) axis was identified as a potential target in liver cancer (LC). Next, we found that DSCR8 is highly expressed in LC cell lines Hep3B and Huh7. In addition, sh-DSCR8 inhibits cell proliferation and promotes cell apoptosis. Furthermore, we certified that DSCR8 serves as function as a sponge for miR-22-3p, while ARPC5 is a target gene of miR-22-3p, and the functions of DSCR8 promoting LC cell proliferation could be rescued by miR-22-3p. This study suggests that lncRNA_DSCR8 promotes LC progression and inhibits its apoptosis by regulating the miR-22-3p/ARPC5 axis, signifying that DSCR8 could be a novel therapeutic target for LC.

## Introduction

Liver cancer (LC) is one of the most common malignant tumors worldwide. According to the analysis of GLOBOCAN 2018, LC is the seventh most frequently occurring cancer in the whole world and the second most common cause of cancer mortality 1. The highest incidence rates of LC in the world are found in Asia and Africa, with China accounting for 47% of incidences 1, 2. Although great efforts had been made to prevent and treat LC, the incidence and mortality of LC are still rising 3. Approximately 70-80% of patients are in unresectable stages when LC is diagnosed, owing to the lack of specific signs and symptoms in the early stages of the disease 4. Although many different medications are used for treatment 5, 6, the prognosis of patients with advanced LC remains poor. Hence, the exploration of new specific molecular mechanisms and potential molecular biomarkers for LC is indispensable for diagnosis and treatment.

Long noncoding RNAs (lncRNAs) are noncoding transcript clusters of more than 200 nucleotides in length. They are distinguished from other small noncoding RNAs, such as lncRNAs, tRNAs, and miRNAs. MicroRNAs (miRs) are a class of small noncoding RNAs about 21-25 nucleotides long, which can induce the suppression or degradation of targeted mRNAs by binding to the 3' untranslated region (UTR) 7. It has been reported that about 30% of human genes can be regulated by miRNAs 8. lncRNAs can affect the expression of various genes by regulating the function of relevant miRNAs 9. The ceRNA hypothesis is based on lncRNA-miRNA and miRNA-mRNA competitive interactions 10. Several studies have shown that lncRNAs are often dysregulated in a great number of malignant tumors, which include LC 11-14. Therefore, finding specific lncRNAs is thought to be an important strategy for early diagnosis and clinical treatment of LC.

Accumulating evidence shows that lncRNA, miRNA, and mRNA are involved in the genesis and development of different types of cancer. Circ-0000105 indirectly upregulated the expression of PIK3R1 by absorbing miR-498, and the increase in circ-0000105 inhibited apoptosis and deteriorated proliferation of LC cells, which was related to the high T stage of LC patients and poor differentiation of the tumor 15. Has-circ-0077210 might serve a momentous therapeutic role in restraining the occurrence and development of LC through a ceRNA network, which is composed of hsa-miR-92b-3p, CPEB3, ACADL, and the upstream regulatory molecule has-circ-0077210 16. The negative relationship between TTN mutation and immune cell infiltration was detected, and TTN mutation predicted a poor prognosis in LC, based on the construction of a ceRNAs network. In addition, TTN played a crucial role in the control and regulation of immune cell infiltration in LC; thus, it could be used as a therapeutic target 17. However, the roles of the ceRNA network in LC, especially the analysis of ceRNA regulatory networks based on large sample size and next-generation sequencing, have not yet been completely expounded.

Gene Expression Omnibus (GEO) and The Cancer Genome Atlas (TCGA) are both public large-scale cancer omics databases. Several thousands of transcriptomic profiles can be used for retrospective analyses from these two public databases 18. In this study, we employed these databases to construct a ceRNA network by bioinformatic analysis and identified a ceRNA axis, i.e., the Down syndrome critical region 8 (lncRNA_DSCR8)/miR-22-3p/actin-related protein 2/3 complex subunit 5 (ARPC5) axis, that could be used as a therapeutic target. Then, a series of in vitro and in vivo experiments were performed to test the regulatory effect of the lncRNA_DSCR8/miR-22-3p/ARPC5 axis in LC cell lines (hep3B and Huh7 cells) and nude mice with Hep3B xenografted tumor.

## Materials and Methods

### GEO data download

We searched the GEO database for data sets related to LC using the keywords "liver cancer." Next, GSE70880, GSE138178, GSE25097, GSE36376, GSE76427, GSE64041, and GSE14811 were selected and downloaded.

For lncRNA expression profiling, a total of 16 LC tissues and paired adjacent noncancerous liver tissues were enrolled in GSE70880 (platform: GPL19748 Agilent-038314 CBC Homo sapiens lncRNA + mRNA microarray V2.0), while 49 LC tissues and paired adjacent noncancerous liver tissues were enrolled in GSE138178 (platform: GPL21827 Agilent-079487 Arraystar Human LncRNA microarray V4).

For mRNA expression profiling, 5 GEO datasets were downloaded. In total, 268 LC tissues and 289 non-LC tissues (243 adjacent non-tumor, 40 cirrhotic, and 6 healthy liver samples) were enrolled in GSE25097 (platform: GPL10687 Rosetta/Merck Human RSTA Affymetrix 1.0 microarray, Custom CDF), and 240 patients with LC tumor liver and 193 patients with non-tumor liver were enrolled in GSE36376 (platform: GPL10558 Illumina HumanHT-12 V4.0 expression beadchip). In addition, 115 patients with LC tumors and 45 patients with adjacent non-tumor liver tissue were enrolled in GSE76427 (platform: GPL10558 Illumina HumanHT-12 V4.0 expression beadchip). A total of 60 LC biopsies and 65 non-tumor liver tissue (60 adjacent non-tumor liver biopsies and five normal liver biopsies) were enrolled in GSE64041 (platform: GPL6244 Affymetrix Human Gene 1.0 ST Array), while pair-matched tumor and adjacent liver tissues from 56 LC patients were enrolled in GSE14811 (platform: GPL8177 KRIBB_Human_14K).

### DELs/DEmRs screening

First, the differentially expressed lncRNAs (DELs) between LC tissues and normal samples were screened by GEO2R online software (p adjust < 0.05 and | log FC |>1.5). The overlapping lncRNAs in GSE70880 and GSE138178 were selected as DELs. In addition, as for differentially expressed mRNAs (DEmRs), p < 0.05 was selected as the threshold, and the overlapping mRNAs in GSE25097, GSE36376, GSE76427, GSE64041, and GSE14811 were selected as DEmRs.

### Prediction of differentially expressed miRNAs (DemiRs) and mRNAs

We predicted the DEmiRs targeted by DELs and constructed the lncRNA-miRNA pairs based on the correlation data from the miRcode database, based on the DELs. Then, we projected “predicted mRNAs” of DEmiRs by using concurrent data in the miRDB, miRTarBase, and TargetScan databases. The mRNAs present in all three databases were regarded as “predicted mRNAs” of these DEmiRs.

### DEmRs selection

By comparing “predicted mRNAs” with mRNAs from five GEO datasets, only the overlapping mRNAs were used to construct the ceRNA network with DELs and DEmiRs.

### ceRNA network construction

As we know, lncRNA-miRNA pairs and miRNA-mRNA pairs can form lncRNA-miRNA-mRNA pairs. MiRNA can bind to targeted mRNA to promote the degradation of mRNA, while lncRNA can interact with targeted miRNA to inhibit the degradation of mRNA. Herein, we used the R-alluvial package to construct lncRNA-miRNA-mRNA pairs through miRcode (Version 11; http://www.mircode.org/mircode/), miRDB (Version 7.0; http://mirdb.org/), miRTarBase (https://mirtarbase.cuhk.edu.cn), and TargetScan (Version 7.2; http://targetscan.org/vert_72/) based on the DELs, DEMs, and DEmRs.

### GO and KEGG enrichment analysis of DEmRs

To forecast the possible functions of these DEmRs, GO and KEGG pathway analyses of DEmRs were conducted by cluster Profiler R package. As for GO analysis, p < 0.05 was considered statistically significant, and p < 0.05 was the cut-off criteria for KEGG analysis. The GO terms or KEGG terms that matched the criteria were considered significantly enriched terms.

### Construction of the protein-protein interaction (PPI) network

The STRING database (version 11.0, https://string-db.org/) is a public data source that can provide information regarding the interaction between known proteins and predicted proteins. To elucidate the potential protein-protein relationships between DEmRs, a PPI network was constructed using STRING. Interactions with confidence scores above 0.4 were considered significant and were retained. DEmRs with a number of connections ≥ 4 were considered hub genes in the PPI network.

### ceRNA axis selection and correlation analysis

Initially, ceRNA axes based on DEmRs in PPI and KEGG pathways were constructed. Then, we selected ceRNA axes by overlapping these two axes, and survival analysis of these DEmRs was conducted using the Kaplan Meier-plotter website tool (http://kmplot.com/analysis/index.php?p=service&cancer=liver_rnaseq). Moreover, survival analysis of DEmiRs and DELs was performed using survminer package R (version 3.6.3), based on the data from TCGA (https://portal.gdc.cancer.gov/) and the detailed clinic parameters of enrolled patients was shown in Table S1. Considering the subcellular location is a crucial affect factor of lncRNAs function, we analyzed the subcellular localization of the DELs in LNCipedia (https://lncipedia.org/) and lncLocator (http://www.csbio.sjtu.edu.cn/bioinf/lncLocator/). Finally, only ceRNA axes that included DEmiRs and DEmRs with survival and expression differences and DELs with meaningful subcellular location were selected for subsequent analysis.

### Comprehensive function analysis of ceRNA axis

To investigate the expression of the DEmRs in the ceRNA axis, the Human Protein Atlas (HPA) (https://www.proteinatlas.org) was searched in LC cell lines and at the protein level. The mutation status of DemR was searched and download in cBioPortal for Cancer Genomics (http://www.cbioportal.org). To evaluate the diagnostic value of DEmRs in LC, a ROC curve was generated using the pROC package and the data from TCGA.

### Dual-luciferase reporter gene assay

MiRCode and miRDB were used to predict the targeted binding relationship between DSCR8 and miR-22-3p and between miR-22-3p and ARPC5. Sequences of the 3'UTR of DSCR8 and ARPC5, amplified by PCR, were inserted downstream of the luciferase vector pmirGLO (Promega, WI, USA) to establish WT-DSCR8 and WT-ARPC5 constructs. Mutation sites at 3'UTR of DSCR8 and ARPC5 were generated using the QuickChange multi-site directed by mutagenesis kit (Stratagene, La Jolla, CA, USA). And their mutant, (MUT)-DSCR8 and MUT-ARPC5, were then built. Subsequently, these vectors were co-transfected with miR-22-3p mimic/mimic NC into 293T cells. Renilla luciferase expression vector pRL-TK (TaKaRa, Dalian, China) was used as an internal reference. The activity of luciferase was assayed by a dual-luciferase assay kit (Promega, Madison, WI, USA) after 48 h of incubation. The experiment was conducted in triplicate. Next, 293T cells were obtained from Cell Bank (Shanghai, China, Adherent cell, 1x106, RRID: CVCL_0321) and cultured in DMEM (Sigma, Shanghai, China) containing 10% fetal bovine serum (Hyclone, Logan, UT, USA). Cells were maintained in an incubator with 5% CO2 at 37°C. Cells were passaged and cultured when reaching 90% confluence, and the ninth passage was employed in this study.

### Cell treatment and transfection

Human liver cancer cell lines, Hep3B and Huh7, and normal liver cells, LO2, were transfected with si-DSCR8 and corresponding negative controls using Lipofectamine 2000 Reagent (Invitrogen, Carlsbad, CA, USA), according to the manufacturer's protocol. After 4-6 h of transfection, the medium was replaced with fresh medium containing 10% FBS.

### CCK8 assay

The cell viability was determined by a Cell Counting Kit-8 (Houston TX, USA), according to the manufacturer's instructions. In brief, the cells were seeded in a 96-multiwell culture plate with 80% confluence. Subsequently, CCK-8 solution was added to each well and incubated at 37ºC for 1 h. Finally, the absorption at 450 nm was measured using a microplate reader (BioTek, Burlington, USA). The cells viability curve was generated by calculating the mean value and standard deviation of the optical density (OD) for every five wells.

### Flow cytometry assay

To measure the death of LC cells, a flow cytometry assay was conducted using propidium iodide (PI, Sigma Aldrich, Shanghai, China) staining. The cells were prepared in a fluorescence-activated cell sorting (FACS) tube with FACS buffer. To the tube, 10 μL PI was added and incubated for 15 min in the dark. Dead cells were determined by a flow cytometer (BD Bioscience, New York, USA).

### Quantitative real-time reverse transcription-polymerase chain reaction (qRT-PCR)

Total RNA was purified from cells using Trizol reagent (Invitrogen, Carlsbad, CA, USA). Approximately 2 µg RNA was transcribed into complementary DNA (cDNA) using PrimeScript RT Reagent Kit (Invitrogen; Thermo Fisher Scientific, Inc., MA, USA). SYBR Premix Ex Taq™ (Takara Biotechnology Co., Ltd., Dalian, China) was used for qRT-PCR via a Light Cycler 480 (Roche, Indianapolis, IN, USA). All samples were analyzed in triplicate on the same plate, and GAPDH was the internal reference gene. The relative expression levels were calculated using the comparative cycle quantification (2-△△Ct) method.

### Western blotting

After corresponding treatment, LC cells were lysed using RIPA cell lysis buffer (Cell Signaling Technology, Danvers, MA, USA) containing protease inhibitor and phosphatase inhibitor. The cellular protein was extracted and quantified by BCA protein assay kit (Beyotime Institute of Biotechnology, Shanghai, China). The same amount (50 μg) of protein obtained from different samples was separated by SDS-PAGE electrophoresis and transferred to polyvinylidene fluoride (PVDF) membranes. The membranes were reacted with specific primary antibodies and fluorescent secondary antibodies after being blocked in 5% non-fat dry milk solution. The primary antibodies were monoclonal mouse antibodies against ARPC5 (1:1000, Abcam, Cat#ab51243, Cambridge, UK); β-catenin (1:1000, Abcam, Cat#ab32572, Cambridge, UK); cyclin D1(1:1000, Abcam, Cat#ab16663, Cambridge, UK); c-myc (1:1000, Abcam, Cat#ab32072, Cambridge, UK); and β-actin (1:10000, Abcam, Cat#ab8226, Cambridge, UK). The membranes were washed with tris-buffered saline containing 1% tween 20 and reacted with chemiluminescent reagents (Millipore, MA, USA). The respective densities of the protein bands were evaluated by ImageJ software. In this study, the loading control was GAPDH, and the data were expressed as the ratio of specific protein expression to GAPDH expression.

### Plate clone formation assay

The Hep3B and Huh7 cells transfected by lentivirus and untreated cells were plated in 6-well plates (NEST, Hong Kong, China) at a density of 1000 cells per well. The cells were fixed with 4% paraformaldehyde after culture for 15 days and washed with PBS. Finally, the cells were stained with 0.1% crystal violet.

### Tumorigenicity assay in nude mice

Male BALB/c nude mice aged 4 to 5 weeks (Hunan SJA Laboratory Animal Co., Ltd) were housed in a specific pathogen-free (SPF) environment in the Animal Laboratory Unit, Key lab, Tianjin Medical University Cancer Institute and Hospital. Hep3B (n = 5) or Hep3B-siDSCR8 (n = 5) cells were suspended at a concentration of 108/mL in phosphate-buffered saline. The cell suspension was mixed with matrix glue (1:1 v/v) and placed on ice prior to use. At this time, the cell density was 5 × 10^7^ cells/mL. Each nude mouse was injected with 200 μL of the mixture, that is, each nude mouse was inoculated with 1 × 10^7^ cells. Tumor growth was monitored daily, and tumor volume was assessed by measuring the tumor size. The tumor volume was calculated according to V = (length × width × 2)/2. All animal studies were approved by the Yantai Affiliated Hospital of Binzhou Medical University (2018YFC1315601). We followed the guidelines entitled “Laboratory Animal-Guideline for ethical review of animal welfare” of People's Republic of China (GB/T 35892-2018). The difference between each group was calculated by non-paired t-test, and a p value < 0.05 indicates statistical difference. All analyses were performed with Graph Pad Prism 7.0.

### Immunohistochemistry assay

The immunohistochemical staining procedure was conducted according to the kit instructions. The Ki-67 and caspase-3 proteins were immunohistochemically localized in the cytoplasm, as indicated by brown-yellow particles. The entire slice was photographed under an optical microscope to evaluate the positive staining. Five fields were randomly chosen to judge whether Ki-67 and caspase-3 was positively expressed. Under the microscope, the sample was photographed and recorded at ×100 magnification.

### Statistical analysis

In this study, GraphPad Prism was used for data analysis. Data are expressed as the mean ± standard deviation and obtained from at least five separate experiments. The levels of significance of differences were determined by one-way ANOVA among various treatments. A value of p < 0.05 was considered statistically significant.

## Results

### Technical route of this study

**Fig 1:**
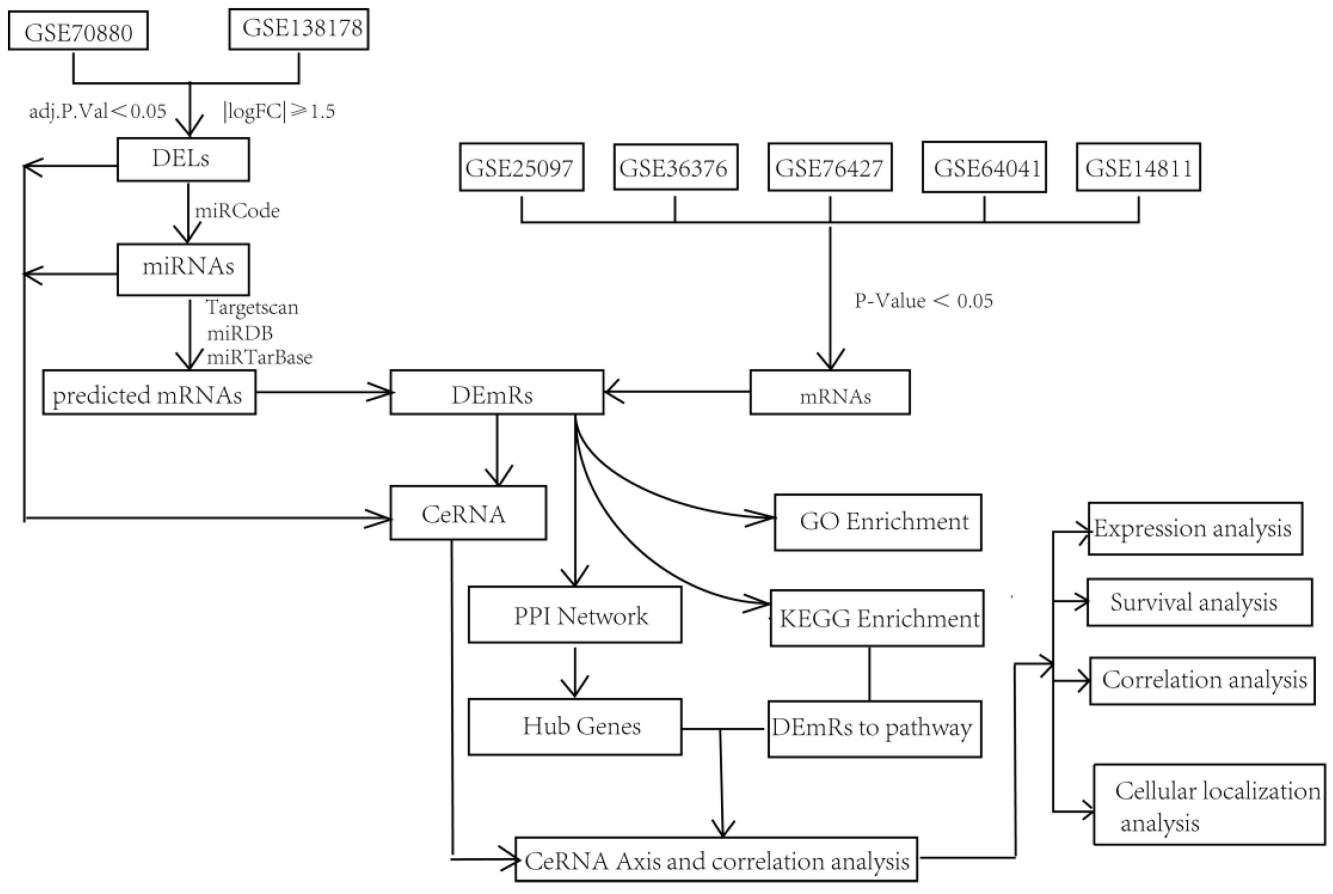
Flowchart of the construction and analysis of a LC-related ceRNA regulatory network.

To increase the accuracy of the prediction analysis, two datasets were selected for the differentially expressed lncRNAs (DELs), and five datasets were selected for the mRNAs from GEO database. Then, DEmiRs were predicted by the use of miRcode, miRDB, miRTarBase, and TargetScan, based on the DELs. Next, mRNAs were predicted based on these DEmiRs. Moreover, these “predicted mRNAs” were overlapped with mRNAs from five different datasets and DEmRs. The ceRNA network was constructed based on the DELs, DEmiRs, and DEmRNAs. To elucidate the possible function of this ceRNA network, GO, KEGG, and PPI analyses were performed on the DEmRNAs. ceRNA axes related to DEmRs in the PPI network and KEGG pathway (p < 0.05) concurrently were selected, and expression analysis, survival analysis, correlation analysis, and subcellular localization analysis were performed for these RNAs. Finally, the DSCR8/miR-22-3p/ARPC5 ceRNA axis was singled out, and the correlation of ARPC5 with pathological and clinical data was investigated (Fig. 1).

### Identification of DELs and DEmRs

**Fig 2:**
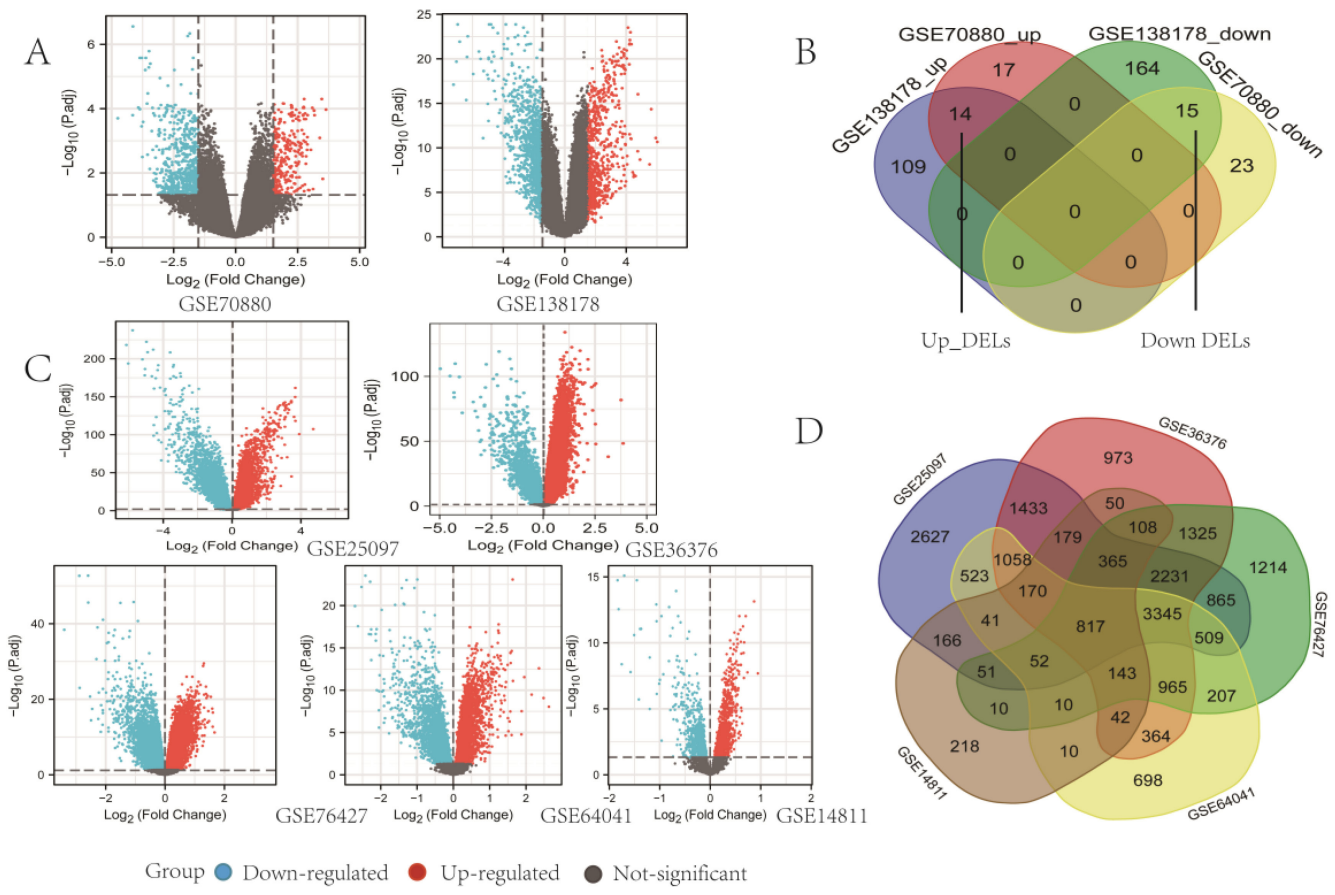
** Identification of differentially expressed lncRNAs (DELs) and mRNAs (DEmRs).** (A) Volcano plots of DELs identified from GEO databases using the GEO2R online software (p adjust < 0.05 and | log FC |>1.5). Red dots denote upregulated lncRNAs; blue dots denote downregulated lncRNAs; and grey dots denote not-significant lncRNAs. (B) Venn diagrams representing the intersections of DELs, the upregulated DELs (Up_DELs) and downregulated DELs (Down_DELs). (C) Volcano plots of DEmRs (p < 0.05). Red dots denote upregulated DEmRs; blue dots denote downregulated DEmRs; and grey dots denote not-significant DemRs. (D) Venn diagrams representing the intersections of DEmRs.

Data from GEO were separately analyzed by lncRNAs and mRNAs, according to different microarray datasets in R software (version 3.6.3). Through screening, we found a total of 29 DELs common to both GSE70880 and GSEGSE138178, among which 14 DELs were upregulated and 15 were downregulated in LC (Fig. 2A, Fig. 2B). By overlapping all 5 mRNAs datasets, we identified 817 mRNAs in GSE25097, GSE36376, GSE76427, GSE64041, and GSE14811 for the next step in the analysis (Fig. 2C, Fig. 2D). A Venn diagram was generated using online software (http://bioinformatics.psb.ugent.be/webtools/Venn/).

### Construction of the ceRNA network and selection of ceRNA axis

**Fig 3:**
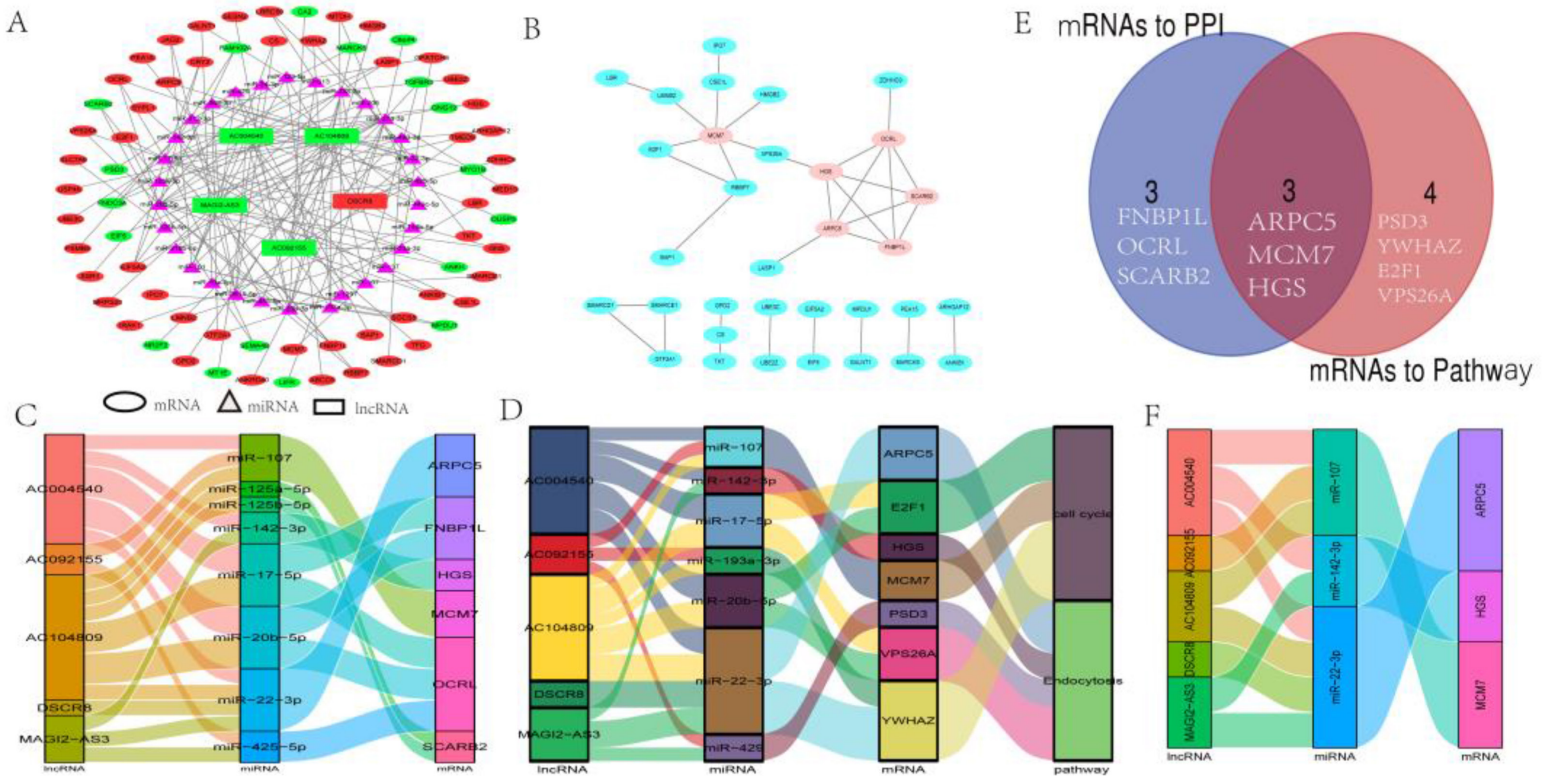
** Construction of the ceRNA and PPI networks and selection of ceRNA axis.** (A) lncRNA-miRNA-mRNA ceRNA network in LC. All the nodes in the network were DEmRs. Red and blue letters indicate differentially expressed genes between non-tumor liver tissue samples and LC. Ovals represent mRNAs. Triangles represent miRNAs. Green rectangles represent downregulated DELs, and red rectangles represent upregulated DELs. (B) The PPI network of DEmRs between non-tumor liver tissue samples versus LC samples. The ovals represent DEmRs, and the brown ovals represent hub genes. (C) Sankey diagram of KEGG pathway-related DEmRs. (D) Sankey diagram of related-hub genes in PPI. (E) Venn diagrams representing the intersections of PPI-related DEmRs and KEGG pathway-related DEmRs. (F) Sankey diagram of overlapping DEmRs from PPI hub genes and KEGG pathway mRNAs. In the Sankey diagrams, first left bar: lncRNA; second left bar: miRNA; third left bar: mRNA; fourth left bar: KEGG pathway. The connection degree of each molecular is visualized based on the size of the rectangle.

As shown in Fig. 3A, a ceRNA network was constructed, including 5 lncRNAs nodes, 30 miRNAs nodes, 68 mRNAs nodes, and 161 edges. The relationships between lncRNA-miRNA and miRNA-mRNA are shown in Table S2. It is worth noting that only one lncRNA (DSCR8) was upregulated in LC, and four (MAGI2-AS3, AC004540, AC104809, AC092155) were downregulated. Eighteen mRNAs were downregulated in LC, and 50 were upregulated (Fig. 3A), which is consistent with the ceRNA mechanism.

To further understand the interaction of the mRNAs in this ceRNA, the PPI network of 33 mRNAs was constructed, and ARPC5, OCRL, HGS, MCM7, FNBP1L, and SCARB2 were closely related to the other mRNAs. These six mRNAs were selected as hub genes (Fig. 3B).

### Functional annotation and enrichment analysis of ceRNA network

**Fig 4:**
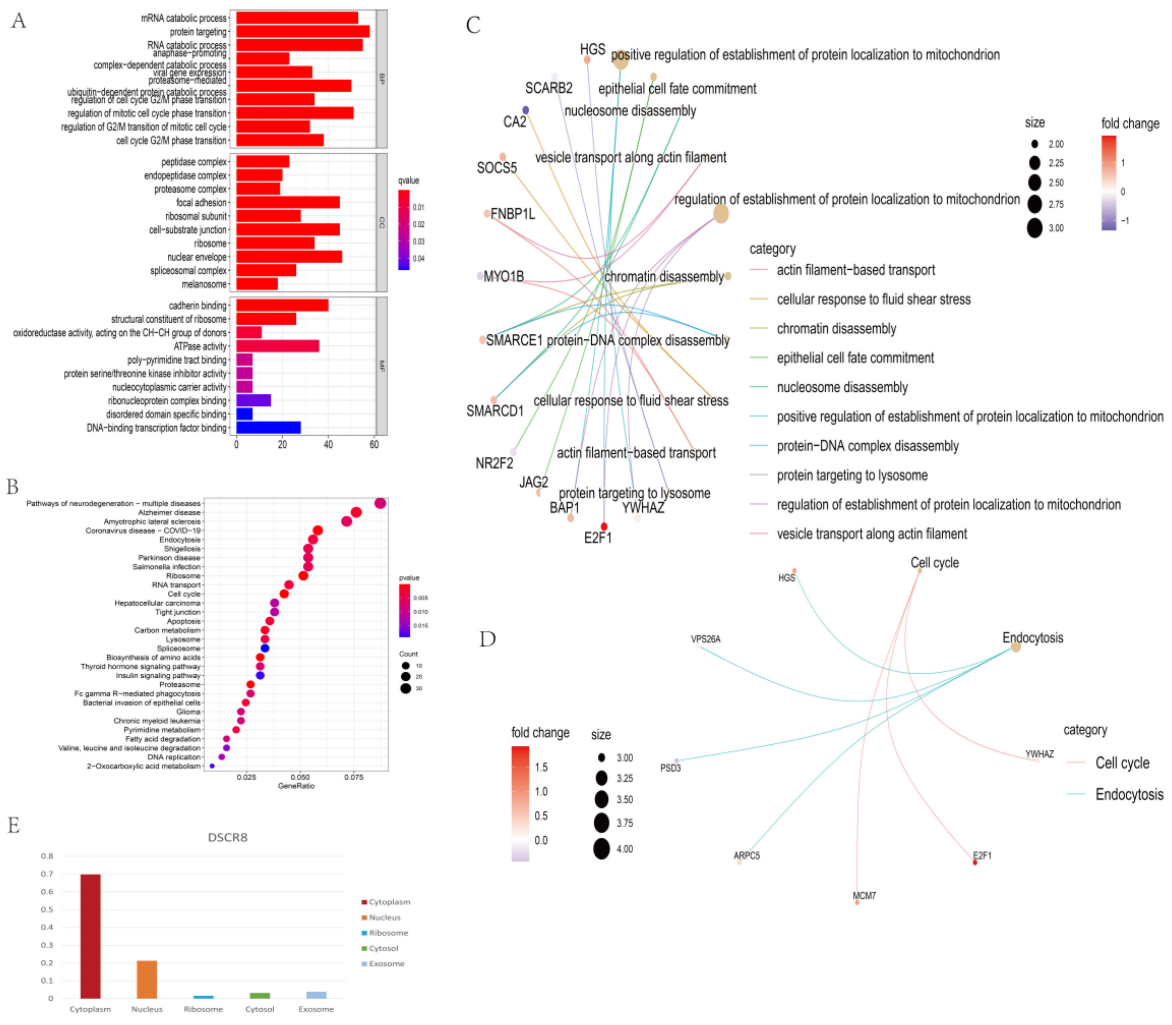
** Functional enrichment analysis of the ceRNA network and subcellular localization analysis of lncRNA.** Top 10 biological process terms (p<0.05) of ceRNA-related DEmRs. (A) GO enrichment analysis of DEmRs between non-tumor liver tissue samples versus LC samples. Bar plot of biological processes (BP); bar plot of cellular component (CC); and bar plot of molecular function (MF). (B) KEGG enrichment analysis of ceRNA network. Top 30 KEGG pathways (p<0.05) of the ceRNA related DEmRs. (C) GO analysis showing the top 10 biological processes of DEmRs of ceRNA. (D) KEGG analysis showing two cell signaling pathways and related DEmRs of ceRNA. (E) The subcellular localization for ARPC5.

GO analysis was conducted from three distinguished aspects: biological process (BP), cell component (CC), and molecular function (MF) for the ceRNA network. As shown in Fig. 4A, Fig. 4C, GO analysis results showed that the potential BP of the mRNAs related to the PPI network was mainly enriched in the positive regulation of the establishment of protein localization to mitochondrion, epithelial cell fate commitment, nucleosome disassembly, and vesicle transport along actin filament. In addition, KEGG analysis revealed only two KEGG pathways were identified as statistically significant at p < 0.05. They were “Cell cycle” and “Endocytosis” (Fig. 4B, Fig. 4D).

### Selection of underlying meaningful ceRNA axis

Initially, ceRNA axes involving two KEGG pathways were constructed, as shown in Fig.3C. Next, ceRNA axes involving PPI hub genes were constructed (Fig. 3D). Since proteins usually need to interact with other proteins to perform their biological functions, proteins that are more closely linked to other proteins and are involved in more cellular functions should be more important in cellular biological functions. Thus, we overlapped mRNAs of six hub genes and mRNAs in KEGG pathways. The overlapped mRNAs, ARPC5, MCM7, and HGS, were selected, and ceRNA axes that incorporated the hub genes were constructed (Fig. 3E and Fig. 3F).

**Fig 5:**
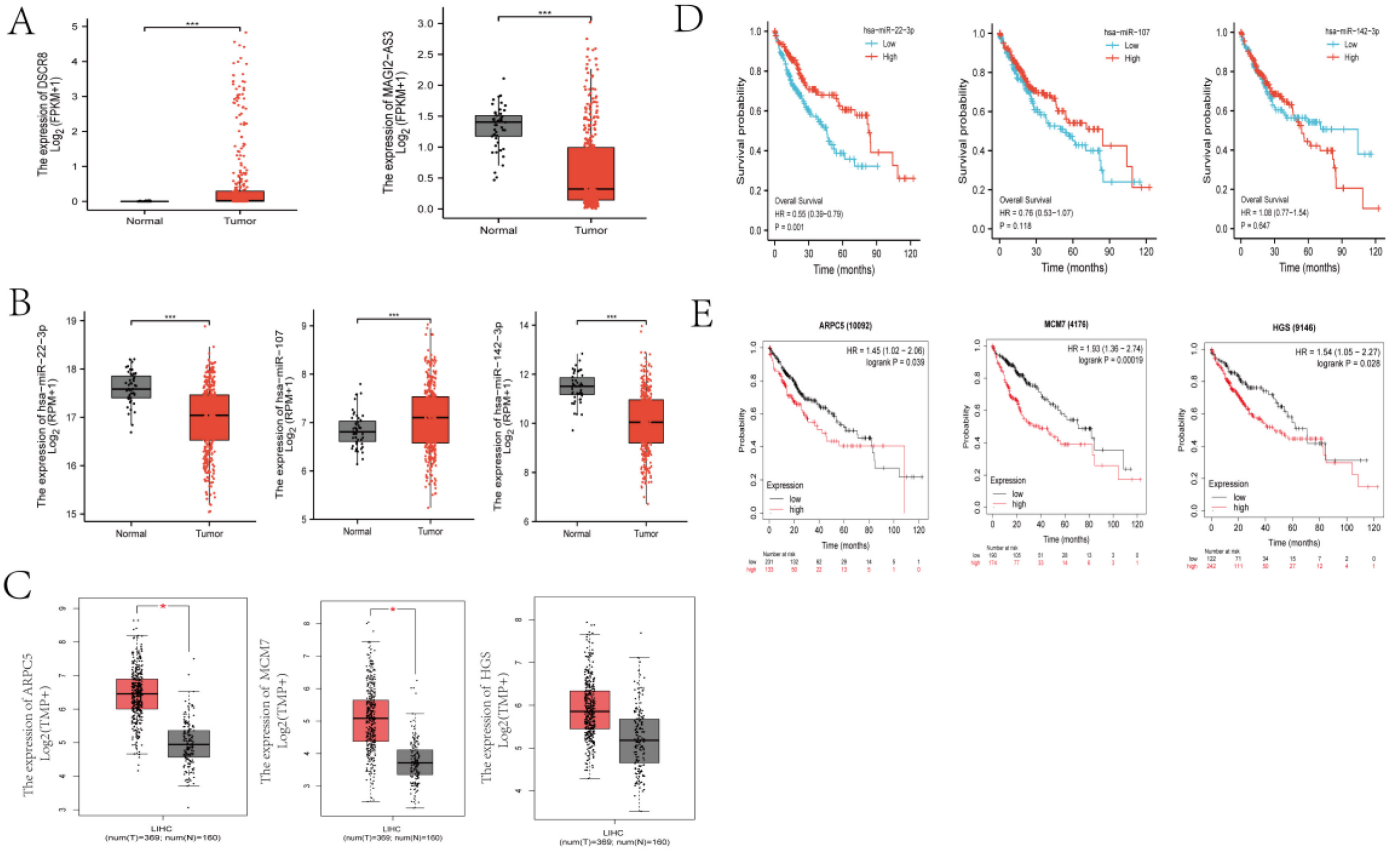
** Distribution and overall survival analysis of RNAs in ceRNA axis.** (A) The distribution of two lncRNAs in the LC tissues and normal tissues (***p<0.001). (B) The distribution of three miRNAs in the LC tissues and normal tissues (***p<0.001). (C) The distribution of three DEmRs in the LC tissues and normal tissues (*p<0.05). (D) Survival analysis of miRNAs were conducted using survminer package R (version 3.6.3) based on the TCGA. (E) Survival analysis of DEmRs. Kaplan-Meier survival curves for selected DEmRs based on TCGA and using the kmplot website tool. (D, E) Horizontal axis, overall survival time (months); vertical axis, survival fuction.

To investigate the clinical significance of the molecules in Fig. 3F, we analyzed the expression patterns and survival relevant information of all of the lncRNAs and miRNAs. Among the five lncRNAs, only DSCR8 and MAGI2-AS3 had expression differences between normal liver tissue and LC tissue (Fig. 5 A). Among the three miRNAs, miR-22-3p, miR-107, and miR-142-3p had expression differences between normal and tumor tissues (Fig. 5 B). Among the three DEmRs, HGS had no significant difference between tumor and normal tissues, but ARPC5 and MCM7 showed significant differences (Fig. 5 C). For the associations between the RNAs in Fig. 3F and overall survival of LC patients, one miRNA (miR-22-3p, Fig. 5 D) and one mRNA (ARPC5, Fig. 5 E) were found to be related to prognosis (p < 0.05). miRNAs mainly play cellular biological roles in the cytoplasm; therefore, only lncRNAs existing in the cytoplasm can combine with miRNAs and perform their function. The cellular localization analysis was performed using the lncLocator website. As shown in Fig. 4E, DSCR8 was mainly located in the cytoplasm, but the subcellular location information of MAGI2-AS3 could not be found. In total, we selected the ceRNA axis, DSCR8/miR-22-3p/ARPC5, for the subsequent studies.

### Analysis of core genes of the selected ceRNA axis

**Fig 6:**
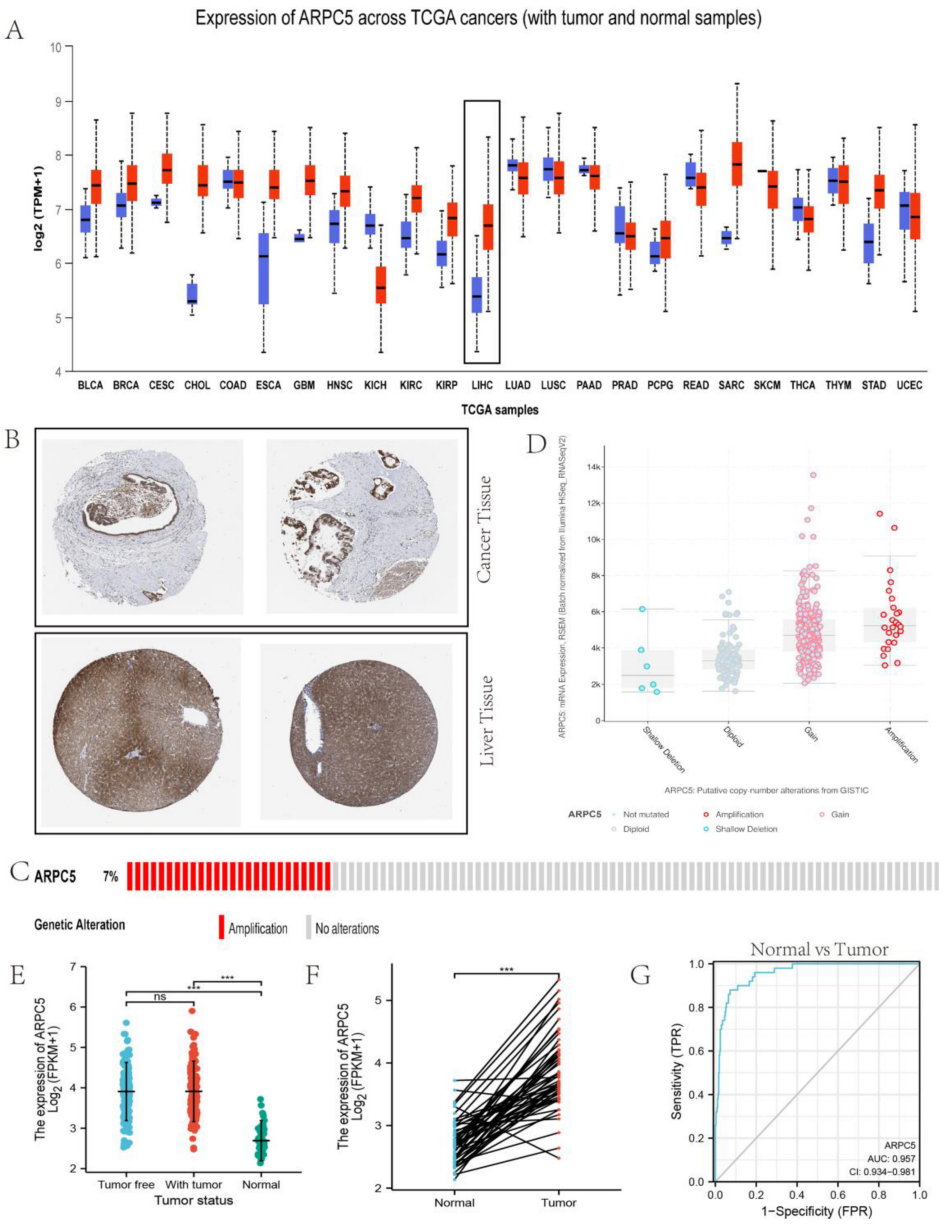
** Role of ARPC5 in LC.** (A) Expression distribution of ARPC5 in pan-cancer samples versus corresponding normal samples. (B) Validation of the expression of ARPC5 on the translational level by the Human Protein Atlas database (immunohistochemistry). The level of ARPC5 protein in LC tissue (lower row) was higher than in normal liver tissue (upper row). (C) The distribution of ARPC5 genomic alterations in TCGA on a cbioPortal OncoPrint plot. (D) The dot plot of association between ARPC5 copy number and mRNA expression. (E) The expression patterns of ARPC5 between normal, tumor, and tumor free samples. (F) The expression patterns of ARPC5 between normal and tumor samples. (G) ROC curve of ARPC5 mRNA expression in normal and LC.

On investigating the possible role of ARPC5 in LC, based on the HPA, we found it was overexpressed in LC tissue but downregulated in normal live tissue (Fig. 6A). Similar results were also demonstrated by immunohistochemistry staining obtained from the HPA (Fig. 6B). As shown in Fig. 6C, the OncoPrint plot showed the amplification of ARPC5 gene in TCGA LC dataset.

Furthermore, LC samples harboring ARPC5 gain and amplification exhibited high mRNA expression, as shown in Fig. 6D. We also found that ARPC5 mRNA expression was significantly higher in LC tissues than in normal tissues, as shown in Fig. 6E and Fig. 6F. However, between tumor free tissues and tumor tissues, the significant difference was based on the data from TCGA database (Fig. 6E). We evaluated the diagnostic value of ARPC5 expression by ROC curve. The results showed that the area under the curve (AUC) of ARPC5 was 0.957.

### Validation of DSCR8/miR-22-3p/ARPC5 axis in vitro

**Fig 7:**
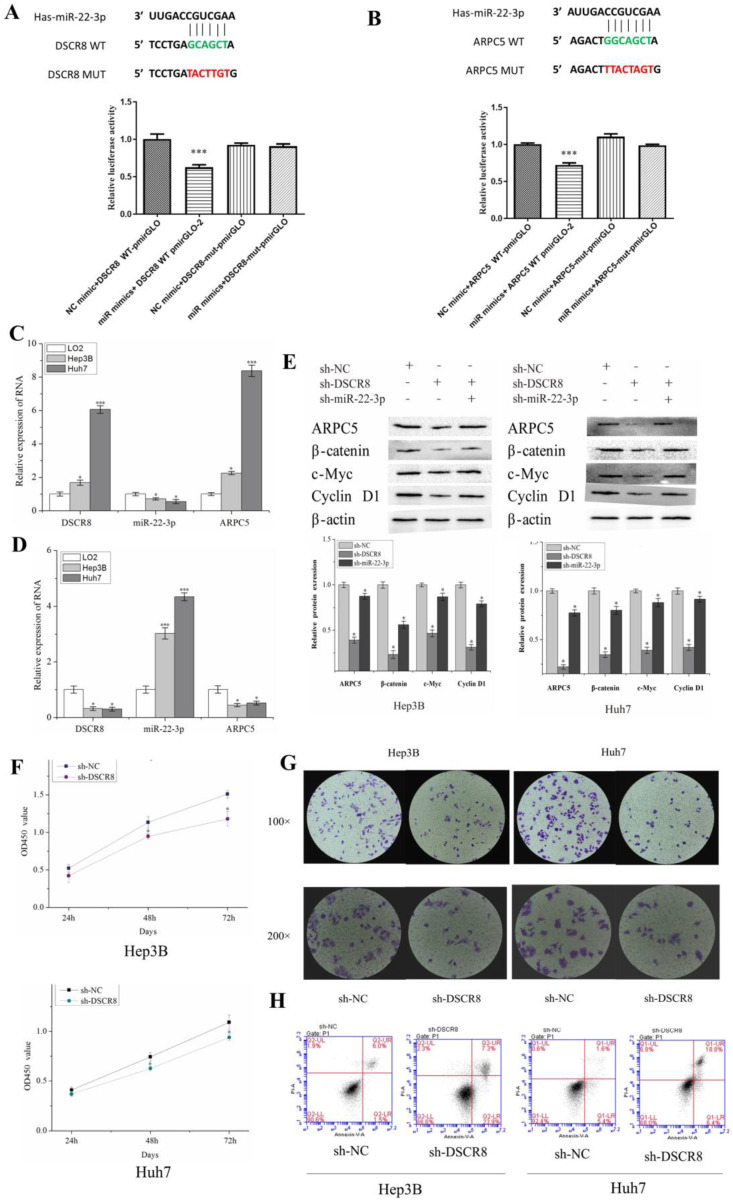
** Validation of DSCR8/miR-22-3p/ARPC5 axis in vitro.** Dual luciferase experiments show miR-22-3p had targeted binding sites with lncRNA DSCR8 (A) and ARPC5 (B). (C and D) The expression of miR-22-3p and ARPC5 were downregulated after DSCR8 inhibition. (E) WB analysis showing the expression of Wnt signaling pathway protein decreased, but the effect could be reversed by the downregulation of miR-22-3p. Cell proliferation (F) and clonal formation (G) were inhibited after knockdown of DSCR8, while apoptosis increased (H).

Dual luciferase experiments showed that miR-22-3p had targeted binding sites with lncRNA DSCR8 (Fig. 7A) and ARPC5 (Fig. 7B), as predicted in the bioinformatics analysis. The results of the dual luciferase reporter assay indicate miR-22-3p mimic reduced the luciferase activity in 293T cells transfected with DSCR8 WT but had no effect on the DSCR8-mut group (Fig. 7A). Furthermore, the dual luciferase reporter assay showed the luciferase activity in miR-22-3p mimics+ ARPC5 WT was apparently weaker than that in other groups (Fig. 7B).

qRT-PCR was used to examine the expression differences of DSCR8, miR-22-3p, and ARPC5 in normal liver cell line (LO2), as well as LC cell lines (Hep3B and Huh7), to observe the relationship between the three (Fig. 7C). Compared to normal liver cell LO2, qRT-PCR showed that DSCR8 and ARPC5 were significantly upregulated in LC cell lines. In addition, miR-22-3p was downregulated in LC cells but upregulated in LO2. The expression of DSCR8 and ARPC5 was negatively correlated with the expression of miR-22-3p.

To explore the function of DSCR8 in LC cells, the expression of miR-22-3p and ARPC5 in LC cell lines was subsequently examined by qRT-PCR, using siRNA to interfere with DSCR8. The results show that after DSCR8 inhibition, miR-22-3p expression was enhanced, but ARPC5 expression was significantly inhibited in LC cell lines (Fig. 7D). Then the expression of ARPC5 protein, as well as β-catenin, a key protein in the Wnt signaling pathway, and downstream target proteins, c-myc and cyclin D1, were detected by Western blot in LC cell lines. After interfering with sh-DSCR8, western blot showed that the expression of ARPC5 protein, β-catenin, c-myc, and cyclin D1 in LC cell lines was inhibited. However, when interfering LC cell lines with sh-DSCR8 and sh-miR-22-3p simultaneously, the expression of ARPC5 protein, β-catenin, c-myc, and cyclin D1 was upregulated (Fig. 7E).

To explore the function of DSCR8 in LC cell lines, we subsequently examined cell proliferation, as well as apoptotic changes, following interference with sh-DSCR8 by CCK8, clonogenic assays, and flow cytometric apoptosis assays. CCK8 and colony formation assays show that Hep3B and Huh7 cell proliferation was significantly inhibited after transfection with sh-DSCR8 (Fig. 7F and Fig. 7G). Flow cytometric apoptosis assays show that the number of apoptotic cells increased after the inhibition of DSCR8 expression (Fig. 7H).

### Validation of DSCR8/miR-22-3p/ARPC5 axis in vivo

**Fig 8:**
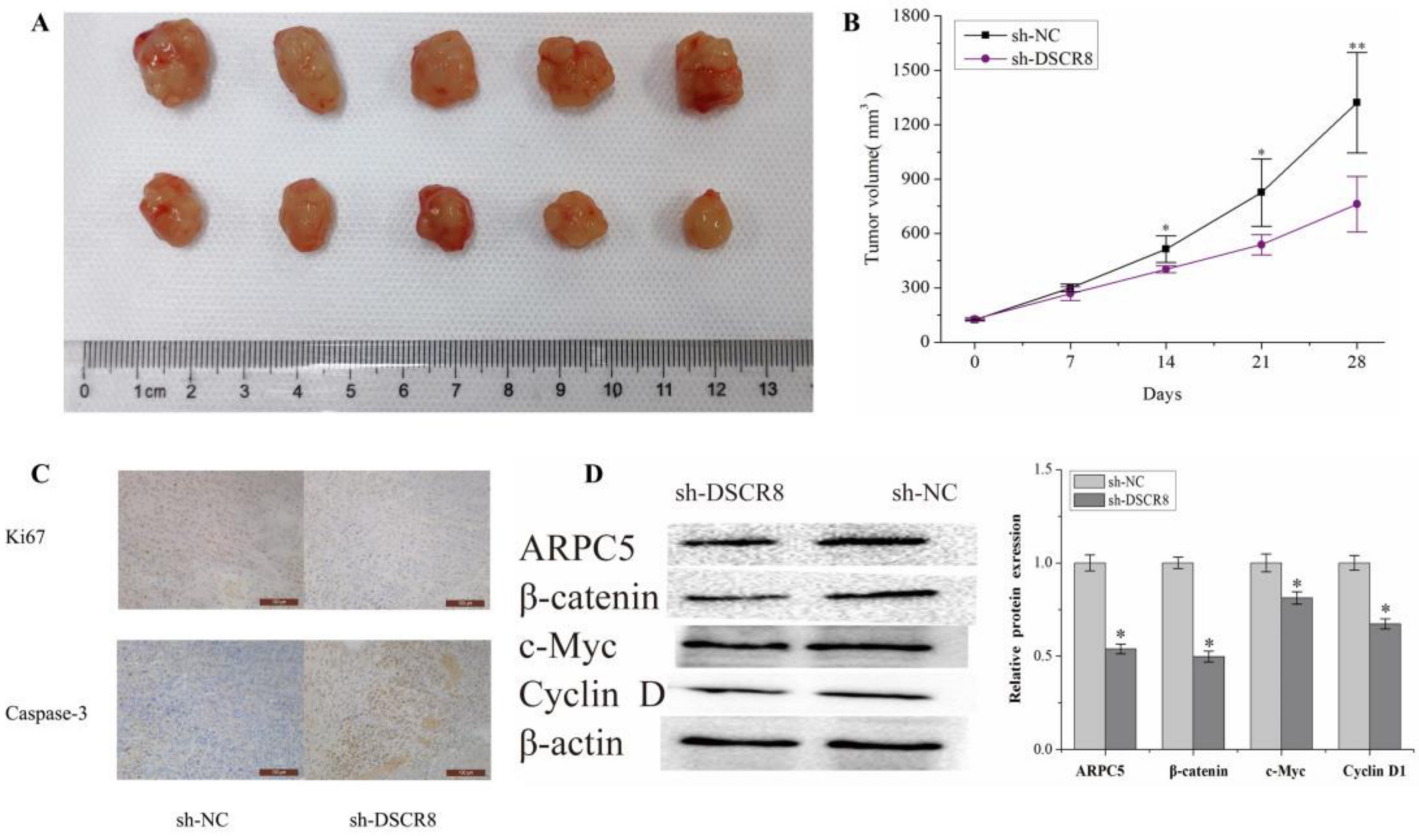
** Validation of DSCR8/miR-22-3p/ARPC5 axis in vivo**. (A) The representative images of tumors over time or with DSCR8 inhibition. The upper row refers to the NC group and the lower row refers to the DSCR8 inhibition group. (B) Data analysis for the tumor volumes after treatment with/without DSCR8 inhibition (*p<0.05, **p<0.01). (C) Immunohistochemistry staining analysis for the expression of Ki-67 and caspase3. (D) Western blot analysis of the Wnt signaling pathway proteins (*p<0.05).

Subcutaneous tumors were established in nude mice by subcutaneous inoculation of Hep3B knockdown DSCR8 and empty Hep3B cells. The tumor volume was observed and measured every week, and the tumor growth curve over 5 weeks was drawn. After 5 weeks, the tumor tissue was killed and weighed. The results show that the volume and the growth rate of the tumor in nude mice decreased significantly after inhibiting the expression of DSCR8 (Fig. 8A and Fig. 8B). The tumor tissue was removed, embedded in paraffin, and sectioned. The expressions of proliferation marker Ki-67 and apoptosis marker caspase-3 were detected by immunohistochemistry. The results show after the expression of DSCR8 was inhibited, the expression of Ki-67 was also significantly inhibited, but the expression of apoptosis marker caspase-3 significantly increased, representing an increase in apoptosis (Fig. 8C). The expressions of ARPC5 protein, β-catenin, c-myc, and cyclin D1 were detected by Western blot. The results are similar to that of the in vitro experiment, in which the expression of several signal pathway proteins was significantly inhibited after knockdown of DSCR8 (Fig. 8D).

## Discussion

LC is one of the most lethal cancers, and hepatocellular carcinoma is the main pathology subtype, which is mainly associated with liver cirrhosis and many other chronic liver diseases 19, 20. According to the World Health Organization's report in 2018, the incidence and mortality of LC was around 840,000 new cases and more than 780,000 deaths each year 1, 21. LC ranked as the fifth most common cause of cancer and the second leading cause of cancer death 22. For advanced LC, many new medications have been developed, such as sorafenib, lenvatinib, regorafenib, cabozantinib, and nivoluma; however, the total five-year overall survival of LC is just 18.1% (95% CI, 17.3%-18.9%) 23-27. Therefore, the investigation of new molecular targets and novel pathogenesis mechanisms for early detection and therapeutic treatment of LC is urgently needed. In this study, we constructed the ceRNA network, selected one ceRNA axis (lncRNA_DSCR8/miR-22-3p/ARPC5), and demonstrated that lncRNA_DSCR8 promoted LC through sponging miR-22-3p to regulate ARPC5. Additionally, we successfully verified this ceRNA network pathway through in vivo and in vitro experiments, proving the relationship between this network and tumorigenesis and development.

PPI networks are typically used to determine which proteins in a network directly interact 28, and hub genes have more opportunities interact with other genes. In our study, a ceRNA network, including 5 lncRNAs, 30 miRNAs, and 68 mRNAs, was first created based on the data from GEO database. Then, a PPI network was constructed with 33 mRNAs, and 6 mRNAs were selected as hub genes with no less than 4 interactions with other genes. We noted that some genes with high combined scores, such as FNBP1L, HGS, DCARB2, ARPC5, OCRL, and MCM7, were high-grade relevance genes in the PPI network. Next, we established a LC-related ceRNA network, based on selected ceRNA axes that involve PPI hub genes and KEGG pathway and have three mRNAs in common (MCM7, HGS, and ARPC5). These ceRNA axes are likely involved in the molecular mechanism of LC pathogenesis and were worthy of further investigation.

To improve the credibility and certainty of our research, we screened all of the selected ceRNA axes. Minichromosomal maintenance protein7 (MCM7) was downregulated in LC mouse models, resulting in tumorigenesis and distant metastasis suppression^28^. MCM7 may promote cell proliferation and silence apoptosis of LC by regulating the PI3K/AKT pathway 29. However, in our study, the upstream expression of miR-107 did not show a significant correlation with LC survival. Moreover, lncRNAs related to miR-107 have not been reported in previous studies. Therefore, we abandoned miR-107/MCM7 as the main ceRNA axis in LC. In addition, the expression of hepatocyte growth factor-regulated tyrosine kinase substrate (HGS) was not significantly different between LC and normal liver tissue, and the upstream expression of miR-142-3p was not correlated with overall survival. Hence, the miR-142-3p/MCM7 axis was also ruled out.

ARPC5 is a subunit of actin-related protein 2/3 complex (Arp2/3). It is widely reported that Arp2/3 plays a crucial role in promoting the proliferation and invasion of various cancers. In head and neck squamous cell carcinoma (HNSCC), the expression of ARPC5 was significantly higher in cancerous tissues than in non-cancerous tissues. MiR-133a can suppress the expression of ARPC5 and inhibit apoptosis and invasion of HNSCC cells 30. In lung squamous cell carcinoma, the expression of ARPC5 was upregulated, and this effect can be repressed by miR-133a 31. In multiple myeloma (MM) cells, ARPC5 expression increased, and high expression of ARPC5 was associated with poor overall survival in MM patients 32. In melanoma, ARPC5 can be a specific target of Yes-associated protein 1 (YAP) and promote melanoma cell progression, survival, and invasion 33. Importantly, ARPC5 can promote LC development through multiple cancer-related pathways and be identified as an independent prognostic biomarker of patient survival 34.

We also obtained similar results showing ARPC5 was upregulated in LC, and high expression of ARPC5 was associated with poor overall survival in LC patients. Based on the HPA database, we verified ARPC5 was overexpressed, and its proteins were also upregulated in LC tissues. The data from cBioPortal confirmed that ARPC5 was amplified in LC. LC samples harboring ARPC5 gain and amplification exhibited higher mRNA expression. ROC analysis revealed that ARPC5 is a potential biomarker for the diagnosis of LC. The above results enhance the feasibility of ARPC5 as a research RNA target.

In our study, miR-22-3p can regulate the expression of ARPC5 by binding to it. Zhao et al. found that the proliferative, invasive, and migratory abilities of Huh7 and LM3 cells were inhibited when miR-22-3p was upregulated, and these effects were achieved through regulating the miR-22-3p/MTA3 signaling pathway 35. Another study showed that miR-22-3p suppressed LC tumorigenesis via the SAL-miat/miR-22-3p/ sirt1 axis 36. Chen et al. reported the β-catenin/ miR-22-3p/TET2 regulatory axis controlled the pathogenesis in alcohol-related LC malignancy 37. The MALAT1/miR-22-3p/IAPs, MIR22HG/miR-22-3p/HMGB1, miR-22-3p/SP1, and MIR4435-2HG/miR-22-3p/YWHAZ axes were also shown to play a role in the molecular mechanism of LC 38-41. After careful review of these studies, we concluded that miR-22-3p was closely related to LC.

For miR-22-3p, lncRNA_DSCR8 is its upstream lncRNA. In ovarian cancer, upregulating lncRNA_DSCR8 promoted cell proliferation, invasion, and EMT and inhibited apoptosis by regulating miR-98-5p 42. In LC, lncRNA_DSCR8 promoted tumor cell progression by regulating the lncRNA_DSCR8/miR-485-5p/FZD7 axis 43, which was different from our ceRNA axie. Subcellular localization analysis showed DSCR8 mainly distributed within the cytoplasm. This provides a basis for its interactions with miRNAs. Moreover, in our triple molecular ceRNA axis, the selected miRNA (miR-22-3p) and mRNA (ARPC5) are both related to LC patient survival, and the lncRNA (DSCR8) exhibited differential expression between normal and LC tumor tissues. In our study, we predicted lncRNA_DSCR8-miR-22-3p-ARPC5 axis by bioinformatic way, and then proved this theory by experiments in vitro and in vivo. Together, these results predict that the lncRNA_DSCR8-miR-22-3p-ARPC5 axis is a regulating mechanism in the occurrence and development of LC and a potential therapeutic target.

It is worth mentioning how our study differs from previous studies in that we first linked DSCR8 with ARPC5 and added tumor suppressor miRNA, miR-22-3p, to make the axis complete. We verified its regulatory network through a series of bioinformatics analyses and dual luciferase experiments. Our results suggest this ceRNA pathway may provide new insight into the potential molecular mechanism of LC carcinogenesis as a new cancer regulatory pathway. However, there remain some limitations in the current study. For example, the in-depth mechanism of the DSCR8/miR-22-3p/ARPC5 network remains to be elucidated. Moreover, clinical experiments on the function and mechanism of this ceRNA axis should be performed in LC.

In conclusion, DSCR8 was overexpressed in LC cell lines, which promoted LC cell proliferation and inhibited apoptosis. In addition, DSCR8 acted as a molecular sponge of miR-22-3p that was overexpressed in LC cells. In addition, ARPC5 was a downstream molecular target of DSCR8/miR-22-3p in LC cells. This work provides new insights into the underlying molecular mechanisms of pathogenesis of LC and identifies DSCR8/miR-22-3p/ARPC5 as a novel, potential therapeutic target for future research.

## Supplementary Material

Supplementary tables.
