# Supplementary material for: Long noncoding RNA DSCR8 promotes the proliferation of liver cancer cells and inhibits apoptosis via the miR-22-3p/ARPC5 Axis
Source: J Cancer. 2023 Jan 1;14(1):35–49. doi: 10.7150/jca.79475 (PMC9809336; doi:10.7150/jca.79475)
Supplement: Supplementary file 1 — Supplementary tables. [file jcav14p0035s1.pdf]

Table S1. The clinical information of TCGA-LIHC

Clinic parameters		
Age		65.68±10.76
Gender	Female	156
	Male	282
Stage	Stage I	57
	Stage II	129
	Stage III	182
	Stage IV	43
	unknow	27
T	T1	22
	T2	90
	T3	197
	T4	119
	TX	10
N	N0	130
	N1	117
	N2	85
	N3	87
	Nx	19
M	M0	387
	M1	30
	MX	21

Table S2 Relations of moleculars in the ceRNAs network

The relations of lncRNAs and miRNAs		The relations of miRNAs and mRNAs	
lncRNA	miRNA	miRNA	mRNA
DSCR8	miR-137	miR-17-5p	ANKIB1
DSCR8	miR-146b-5p	miR-206	TKT
DSCR8	miR-22-3p	miR-363-3p	MYO1B
DSCR8	miR-27a-3p	miR-27a-3p	GNG12
DSCR8	miR-338-3p	miR-20b-5p	LASP1
DSCR8	miR-129-5p	miR-22-3p	YWHAZ
AC004540	miR-212-3p	miR-20b-5p	FAM102A
AC004540	miR-142-3p	miR-22-3p	ARPC5
AC004540	miR-17-5p	miR-20b-5p	E2F1
AC004540	miR-20b-5p	miR-613	FNDC3A
AC004540	miR-761	miR-27a-3p	EIF5A2
AC004540	miR-3619-5p	miR-33a-3p	LMNB2
AC004540	miR-22-3p	miR-1297	GTF2A1
AC004540	miR-23b-3p	miR-107	MCM7
AC004540	miR-1297	miR-429	BAP1
AC004540	miR-27a-3p	miR-761	SOCS5
AC004540	miR-107	miR-20b-5p	ANKH
AC004540	miR-338-3p	miR-23b-3p	LBR
AC004540	miR-449c-5p	miR-3619-5p	TMED9
AC004540	miR-490-3p	miR-107	TGFBR3
AC104809	miR-761	miR-142-3p	MARCKS
AC104809	miR-3619-5p	miR-3619-5p	CS
AC104809	miR-22-3p	miR-17-5p	CRY2
AC104809	miR-24-3p	miR-142-3p	SYPL1
AC104809	miR-363-3p	miR-429	PSD3
AC104809	miR-27a-3p	miR-363-3p	GTF2A1
AC104809	miR-107	miR-27a-3p	EIF5
AC104809	miR-449c-5p	miR-455-5p	IPO7
AC104809	miR-125a-5p	miR-125b-5p	EIF5A2
AC104809	miR-125b-5p	miR-20b-5p	SEMA4B
AC104809	miR-10a-5p	miR-17-5p	FNBP1L
AC104809	miR-455-5p	miR-490-3p	SMARCD1
AC104809	miR-17-5p	miR-3619-5p	MPDU1
AC104809	miR-20b-5p	miR-107	SMARCE1
AC104809	miR-193a-3p	miR-216b-5p	ZDHHC9
AC104809	miR-338-3p	miR-429	GPATCH8
AC092155	miR-107	miR-1297	MTDH
AC092155	miR-33a-3p	miR-20b-5p	ANKIB1
AC092155	miR-425-5p	miR-17-5p	EIF5A2
AC092155	miR-338-3p	miR-3619-5p	JAG2
AC092155	miR-193a-3p	miR-20b-5p	CRY2
AC092155	miR-206	miR-20b-5p	VPS26A
AC092155	miR-613	miR-761	JAG2
AC092155	miR-429	miR-125b-5p	SLC7A6
AC092155	miR-490-3p	miR-27a-3p	TGFBR3
MAGI2-AS3	miR-212-3p	miR-24-3p	SSR1
MAGI2-AS3	miR-137	miR-27a-3p	NR2F2
MAGI2-AS3	miR-142-3p	miR-17-5p	ANKH
MAGI2-AS3	miR-429	miR-27a-3p	ANKRD40
MAGI2-AS3	miR-761	miR-17-5p	LASP1
MAGI2-AS3	miR-3619-5p	miR-20b-5p	RBBP7
MAGI2-AS3	miR-216b-5p	miR-142-3p	TFG
MAGI2-AS3	miR-22-3p	miR-17-5p	RBBP7
MAGI2-AS3	miR-23b-3p	miR-20b-5p	GNS
MAGI2-AS3	miR-363-3p	miR-17-5p	ARHGAP12

MAGI2-AS3	miR-27a-3p	miR-27a-3p	C8orf4
MAGI2-AS3	miR-33a-3p	miR-17-5p	SEMA4B
MAGI2-AS3	miR-425-5p	miR-206	LRRC59
MAGI2-AS3	miR-455-5p	miR-129-5p	GALNT1
MAGI2-AS3	miR-129-5p	miR-20b-5p	OCRL
MAGI2-AS3	miR-490-3p	miR-33a-3p	GPATCH8
		miR-17-5p	VPS26A
		miR-23b-3p	MARCKS
		miR-613	LRRC59
		miR-17-5p	UBE3C
		miR-338-3p	MRPS23
		miR-206	GPD2
		miR-24-3p	MT1E
		miR-27a-3p	LIFR
		miR-425-5p	OCRL
		miR-449c-5p	MTDH
		miR-193a-3p	YWHAZ
		miR-137	CSE1L
		miR-363-3p	DUSP5
		miR-3619-5p	SOCS5
		miR-363-3p	UBE2Z
		miR-17-5p	FAM102A
		miR-23b-3p	CA2
		miR-23b-3p	SESN2
		miR-212-3p	PEA15
		miR-125b-5p	SCARB2
		miR-206	FNDC3A
		miR-20b-5p	ARHGAP12
		miR-27a-3p	USP46
		miR-142-3p	PSMB5
		miR-146b-5p	IRAK1
		miR-17-5p	OCRL
		miR-20b-5p	EIF5A2
		miR-761	CS
		miR-129-5p	ABCC5
		miR-17-5p	E2F1
		miR-125a-5p	SCARB2
		miR-142-3p	FNDC3A
		miR-761	MPDU1
		miR-20b-5p	FNBP1L
		miR-27a-3p	MED13
		miR-142-3p	HGS
		miR-17-5p	GNS
		miR-23b-3p	HMGB2
		miR-10a-5p	GALNT1
